# Perioperative considerations in the paediatric patient with congenital and acquired coagulopathy

**DOI:** 10.1016/j.bjao.2024.100310

**Published:** 2024-09-23

**Authors:** Gabor Erdoes, Susan M. Goobie, Thorsten Haas, Andreas Koster, Jerrold H. Levy, Marie E. Steiner

**Affiliations:** 1 Department of Anaesthesiology and Pain Medicine, Inselspital, Bern University Hospital, University of Bern, Bern, Switzerland; 2 Department of Anesthesiology, Critical Care & Pain Medicine, Boston Children's Hospital, Harvard Medical School, Boston, MA, USA; 3 Department of Anesthesiology, College of Medicine, University of Florida, Gainesville, FL, USA; 4 Institute of Anaesthesiology and Pain Therapy, Heart and Diabetes Centre NRW, Ruhr University Bochum, Bad Oeynhausen, Germany; 5 Departments of Anesthesiology, Critical Care, and Surgery, Duke University School of Medicine, Durham, NC, USA; 6 Divisions of Critical Care and Hematology/Oncology, Department of Pediatrics, University of Minnesota, Minneapolis, MN, USA

**Keywords:** bleeding, bleeding disorders, haemostasis, surgery, tranexamic acid, transfusion, trauma, viscoelastic testing

## Abstract

Neonates, infants, and children undergoing major surgery or with trauma can develop severe coagulopathy perioperatively. Neonates and infants are at highest risk because their haemostatic system is not fully developed and underlying inherited bleeding disorders may not have been diagnosed before surgery. Historically, laboratory coagulation measurements have been used to diagnose and monitor coagulopathies. Contemporary dynamic monitoring strategies are evolving. Viscoelastic testing is increasingly being used to monitor coagulopathy, particularly in procedures with a high risk of bleeding. However, there is a lack of valid age-specific reference values for diagnosis and trigger or target values for appropriate therapeutic management. A promising screening tool of primary haemostasis that may be used to diagnose quantitative and qualitative platelet abnormalities is the *in vitro* closure time by platelet function analyser. Targeted individualised treatment strategies for haemostatic bleeding arising from inherited or acquired bleeding disorders may include measures such as tranexamic acid, administration of plasma, derived or recombinant factors such as fibrinogen concentrate, or allogeneic blood component transfusions (plasma, platelets, or cryoprecipitate).

Herein we review current recommended perioperative guidelines, monitoring strategies, and treatment modalities for the paediatric patient with a coagulopathy. In the absence of data from adequately powered prospective studies, it is recommended that expert consensus be considered until additional research and validation of goal-directed perioperative bleeding management in paediatric patients is available.

## Summary

Paediatric patients undergoing major surgery or with trauma might experience critical bleeding secondary to an associated coagulopathy. The aetiology, characteristics, and management strategies differ from adults generally because of: (1) the maturation of the haemostatic system, (2) previously unrecognised haematological disorders, (3) different mechanisms of illness and injury, and (4) shorter longitudinal accumulation of comorbidities. The lack of validated laboratory testing in the paediatric population makes it difficult to harmonise clinical practice. In addition, the relatively small number of paediatric patients experiencing clinically relevant bleeding in the perioperative period limits the design and conduct of robust clinical efficacy and safety studies, resulting in wide variability in clinical practice, mainly extrapolated from adult protocols.

This review is intended to provide a brief overview of the coagulation system with particular reference to developmental haemostasis and identification of inherited haemostatic disorders, as both/either may potentially impact recognition and management of additional acquired haemostatic disorders in the paediatric patient with deranged coagulation. Feasible approaches for screening paediatric patients with haemostatic disorders are reviewed, and evidence-based options for the management of coagulopathy in the perioperative setting in cardiac, major noncardiac surgery, and trauma are presented. Finally, the fundamentals for the use of modern viscoelastic testing (VET) to guide haemostatic bleeding management and goal-directed therapy in paediatric patients and remaining knowledge gaps are discussed.

## Developmental haemostasis

Haemostasis is an active dynamic process involving procoagulant processes that regulate fibrin clot formation and anticoagulant processes that regulate clot dissolution in the vasculature. Developmental haemostasis describes the maturation of the neonatal and infantile haemostatic system. Blood coagulation is a complex process in which primary haemostasis essentially arises from the interaction between the injured endothelium, von Willebrand factor (vWF), and platelets. The major phases of primary haemostasis include platelet activation, adhesion, and aggregation.^1^ According to the modern cell-based concept of blood coagulation, in contrast to the classical waterfall model of coagulation factor activation, secondary haemostasis is a complex interaction between cells and coagulation proteins, primarily platelets, activated factor (F) VII/tissue factor complex, and thrombin. This interaction occurs in three overlapping phases known as: (1) initiation, which occurs on a tissue factor-bearing cell; (2) amplification, which involves the transformation of the procoagulant stimulus from the initiation phase into platelets and cofactor activation to promote large-scale thrombin formation; and (3) propagation, which entails the formation of substantial amounts of thrombin on the surface of the activated platelet.^2^ Coagulation is accompanied by fibrinolysis, which, along with antithrombin, protein C, and S, is important in the maintenance of the haemostatic balance. Alterations in fibrinolytic capacity can lead to bleeding and thrombosis disorders.^3^

**Fig 1:**
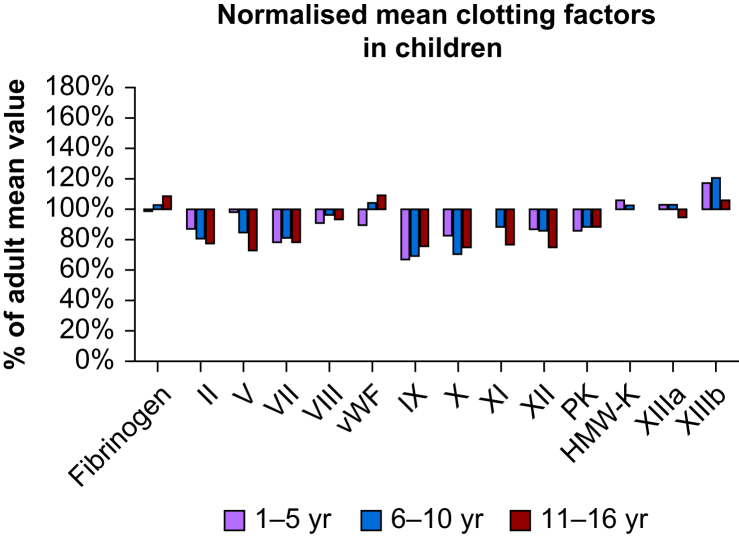
Elements of developmental haemostasis: initial newborn levels compared with adults. Changes in procoagulant factor concentrations in children 1–16 yr of age relative to those in adult. Normalised values calculated as (mean for age)/(mean for adult). Data based on Andrew M, Vegh P, Johnston M, Bowker J, Ofosu F, Mitchell L. Maturation of the hemostatic system during childhood. *Blood* 1992; **80**: 1998–2005. Figure cited from Achey and colleagues.^4^

Haemostatic maturation maintains and variably impacts the delicate balance between bleeding and clotting, including both primary and secondary haemostasis and fibrinolysis (Fig. 1).^4^ The haemostatic system at birth is immature; neonates are born with low concentrations of procoagulant factors, such as vitamin K-dependent factors, and low concentrations of anticoagulant factors that functionally balance haemostasis.^5^ Although bleeding times, as measures of primary haemostasis, in children are longer until they reach the age of 10 yr, children are generally not at increased risk of haemorrhage compared with adults and are not as likely to develop thromboses.^6^ Screening and diagnosis of inherited and acquired haemostatic abnormalities in the paediatric patient must be interpreted using age-appropriate reference ranges and clinical signs and symptoms.

## Inherited or acquired haemostatic disorders

In younger children, the likelihood that a clinically significant but rare inherited haemostatic disorder may not be diagnosed before surgery is higher than in adults, who have had the opportunity for more haemostatic challenges over their longer lifetime. Table 1 provides an overview of inherited disorders associated with coagulopathy, including the haemostatic defect, screening, and laboratory tests helpful for diagnosis. Some disorders of the coagulation system are associated with specific paediatric congenital syndromes and often accompanied by cardiac malformations, orthopaedic malformations (e.g. Noonan, Ehlers–Danlos, and Marfan syndromes) or both.^7^

**Table 1:** Overview of inherited syndromes associated with coagulopathy. aPTT, activated partial thromboplastin time; ATIII, antithrombin; CBC/D/Plt, complete blood count, differential, platelet count; EM, electron microscopy; F, coagulation factor; GP, glycoprotein; INR, international normalised ratio; MDS, myelodysplasia; PFA-100, *in vitro* closure time in platelet function analyser 100; VET, viscoelastic testing; vWD, von Willebrand disease; vWF, von Willebrand factor. Studies within parentheses indicate possible additional tests.

Disorder	Prevalence/genetics	Primary pathophysiology	History/suspicion on questionnaire	Screening/laboratory tests	Diagnosis with special laboratory tests (per haematology)
**Platelets**					
22q11 deletion syndromes (e.g. DiGeorge)	1:2000–1:4000	vWD-like abnormalities Bernard–Soulier-like platelet dysfunction Antibody-mediated thrombocytopenia	Mucosal bleeding Epistaxis Gastrointestinal bleeding Menorrhagia Ecchymoses Bleeding with cuts	CBC/D/Plt INR normal aPTT variable Fibrinogen normal PFA-100 variable	vWD panel Peripheral smear Platelet volume Platelet aggregation (Antiplatelet antibodies) (Platelet EM) (Platelet crossmatching)
Chromosomal aneuploidy	Trisomy 21 - 1:660 Trisomy 18 - 1:5000 Trisomy 13 - 1:5000 Turner - 1:2500	Thrombocytopenia	Mucosal bleeding Petechiae associated with - MDS/leukaemia (Trisomy 21) - Gastrointestinal bleeding (Turner)	CBC/D/Plt INR normal aPTT normal Fibrinogen normal PFA-100 variable	Peripheral smear Platelet aggregation (Platelet EM) (Flow cytometry)
**Combined**					
RASopathies (Noonan's, NF1, Costello, LEOPARD)	Noonan 1:1000–2500	FV, FVII, FX, FXIII Platelet dysfunction	Haematoma Epistaxis Oral ecchymoses Menorrhagia	CBC/D/Plt INR variable aPTT variable Fibrinogen normal PFA-100 variable	Clotting factor activities Peripheral smear Platelet aggregation (VET)
Joint hypermobility spectrum disorders (Ehlers–Danlos, osteogenesis imperfecta, Marfan)	Variable	FVIII, FIX, FXI, FXII, FXIII platelet dysfunction	Ecchymoses Mucosal bleeding Post-traumatic bleeding Surgical bleeding	CBC/D/Plt INR normal aPTT variable Fibrinogen normal PFA-100 variable	Clotting factor activities Protein C/S activities ATIII activities vWD panel Platelet aggregation
Alagille syndrome	Autosomal dominant (variable penetrance) 1:30 000	Variable factor deficiencies Fat-soluble vitamin deficiency Acquired vWD Protein C/S, ATIII Hepatic dysfunction Hyperlipidaemia	Ecchymoses Mucosal bleeding Gastrointestinal bleeding Postsurgical bleeding Intracranial haemorrhage	INR variable aPTT variable Fibrinogen variable PFA-100 variable	Clotting factor activities vWD panel Platelet aggregation Protein C/S activities ATIII activity VET
**Disorder**	**Prevalence (incidence in vWD patients) *Inheritance***	**Primary pathophysiology**	**History/suspicion on questionnaire**	**Screening/laboratory tests**	**Diagnosis with special laboratory tests (per haematology)**
**Plasma factors**					
Haemophilia A	1:5000 males *X-linked recessive*	FVIII deficiency from abnormal liver synthesis	Delayed bleeding Joint and muscle bleeding Post-trauma/surgical bleeding Newborn intracranial haemorrhage Family history	INR normal aPTT variable VET variable PFA-100 normal	FVIII activity (vWD panel)
Haemophilia B	1:25 000 males *X-linked recessive*	FIX deficiency from abnormal liver synthesis	Joint and muscle bleeding Post-trauma/surgical bleeding Family history	INR normal aPTT variable VET variable PFA-100 normal	FIX activity (FVIII activity) (Vitamin K factor activities)
Haemophilia C	1:100 000 *Autosomal variable*	FXI deficiency	Asymptomatic: severe CNS bleeding Gastrointestinal bleeding Epistaxis Menorrhagia Post-trauma/surgical bleeding Family history	INR variable PTT variable VET variable PFA-100 normal	FXI activity (aPTT factor activities)
Factor XII	Unknown prevalence *Autosomal dominant*	FXII deficiency	Usually none (may thrombose)	INR normal aPTT long VET normal PFA-100 normal	FXII activity (aPTT Factor activities)
vWD type 1	Overall vWD 0.1–1:100 (70–80%) *Autosomal dominant*	Quantitative vWF deficiency Mild–moderate quantitative deficiency of normal vWF	Asymptomatic: moderately severe Mucosal bleeding Epistaxis Gastrointestinal bleeding Menorrhagia Ecchymosis Family history	INR normal aPTT variable VET normal PFA-100 variable	vWD panel (Platelet aggregation). (Gene testing)
vWF types 2a, 2b, 2M, 2N	Overall vWD 0.1–1:100 (10–20%) *Autosomal dominant (2N:Autosomal recessive)*	Qualitative vWF deficiency Multimer abnormalities Abnormal binding to - Collagen - Platelets - FVIII (deficiency)	Moderate: moderately severe bleeding Mucosal bleeding Epistaxis Gastrointestinal bleeding Menorrhagia Family history	INR normal aPTT variable VET normal PFA-100 variable	vWD panel (Platelet aggregation) (Gene testing) Gel electrophoresis
vWD type 3	Overall vWD 0.1–1:100 (1–3%) *Autosomal recessive*	Nearly complete vWF deficiency Severely low FVIII	Severe bleeding Mucosal bleeding Epistaxis Gastrointestinal bleeding Menorrhagia Joint bleeding Family history	INR normal aPTT long VET variable PFA-100 abnormal	vWD panel (gene testing)
**Platelets**					
Bernard–Soulier	1: 1 000 000 *Autosomal recessive (usually)*	Defect in GP1b-IX-V surface receptor Macrothrombocytopenia Platelet dysfunction	Severe bleeding Mucosal bleeding Epistaxis Gastrointestinal bleeding Menorrhagia Bruising Bleeding with cuts Family history	CBC/D/Plt abnormal INR normal aPTT normal Fibrinogen normal PFA-100 abnormal	Peripheral smear Platelet aggregation Gene expression Platelet EM
Glanzmann thrombasthenia	1: 1 000 000 *Autosomal recessive*	Defect in platelet GP2B or GP3a Poor cross-linking and clot retraction	Severe mucocutaneous bleeding Bruising	CBC/D/Plt normal INR normal aPTT normal Fibrinogen normal PFA-100 abnormal	Peripheral smear Platelet aggregation Gene expression Platelet EM

**Fig 2:**
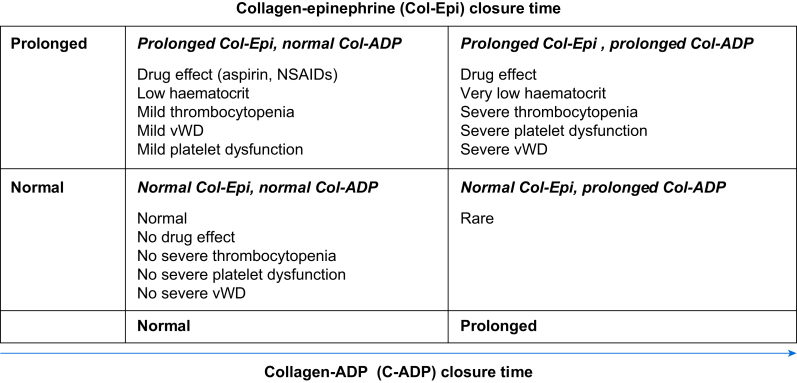
Diagnostic possibilities and interferences of PFA-100 reagent responses. Collagen–epinephrine (Col-Epi) closure time. NSAIDs, non-steroidal anti-inflammatory drugs; PFA-100, platelet function analyser; vWD, von Willebrand disease.

The incidence of acquired bleeding disorders (Table 2) is most prevalent in congenital heart disease, with 37% of preoperative screening tests identifying an imbalance.^8^ Patients with cyanotic heart disease have progressive procoagulant factor imbalance (e.g. FVIII, vWF, increased peak height and endogenous thrombin potential, and a decreased lag time and time-to-peak of whole blood thrombin generation)^9^ as their haematocrit approaches 0.60.^8^ Patients with single ventricle physiology develop progressive right ventricular failure and factor imbalance as their right heart pressures climb, producing chronic liver congestion, dysfunction, and factor losses from protein-losing enteropathy.^10^ In Fontan patients, vitamin K-dependent factors, including protein C, may also be decreased, whereas FVII, antithrombin, and tissue factor pathway inhibitor are increased.^10^ The net haemostatic balance may ultimately be prothrombotic but varies among individuals and disease state, as developmental and genetic considerations may also affect haemostatic balance. High shear stress associated with high blood flow velocities unfolds the vWF large multimers that are susceptible to enzymatic cleavage via metalloproteinases.^11^ Laboratory patterns of this acquired von Willebrand disease (vWD) type 2A and bleeding events are reported in adults and children with aortic valve stenosis, extracorporeal life support, and ventricular assist devices.^11^^,^^12^ A high incidence (63.3%) of acquired vWD is reported in children with congenital heart disease undergoing cardiac surgery.^13^ In this retrospective study of 627 patients, acquired vWD was diagnosed with the following: antigen assays, the platelet function analyser (PFA-100; Siemens Medical Solutions, Malvern, PA, USA) closure time, ristocetin cofactor activity, and in selected cases with gel electrophoresis which evaluates the different multimer fractions. The PFA-100 closure time (which measures quantitative and qualitative platelet abnormalities) prolongation provided the quickest and clearest result in comparison with other variables, thus leading to a rapid diagnosis. However, it has to be considered that a large proportion of patients with congenital heart disease are taking aspirin, which impacts the PFA-100 closure time, particularly the collagen–epinephrine cartridge (Fig. 2). Notably, acquired vWD type 2A has been observed in patients with left and right ventricular outflow tract obstruction and those with atrioventricular or isolated ventricular septal defects. Elimination of the triggering shear points may result in some normalisation of the multimer distribution.^11^^,^^14^ This has to be considered in patients undergoing additional cardiac and noncardiac procedures in the future. The potential of PFA-100 closure time as a screening tool in patients with vWD seems to be promising but needs to be substantiated by further studies.^15^^,^^16^

**Table 2:** Overview of acquired bleeding disorders. aPTT, activated partial thromboplastin time; ATIII, antithrombin; avWD, acquired von Willebrand disease; CBC/D/Plt, complete blood count, differential, platelet count; INR, international normalised ratio; PFA-100, *in vitro* clotting time in platelet function analyser 100; VET, viscoelastic testing; vWD, von Willebrand disease; vWF, von Willebrand factor.

Disorder	Pathophysiology	History/suspicion on questionnaire	Screening/laboratory tests	Diagnosis with special laboratory tests (per haematology)	Comments
**Plasma factors**					
avWD	Similar to type 2 vWD Abnormalities in vWF as a result of abnormal sheer Abnormal endothelial proliferation Platelet dysfunction	Epistaxis Mucosal bleeding Gastrointestinal bleeding Menorrhagia Bruising	INR normal aPTT normal PFA-100 abnormal	vWD panel Platelet aggregation	May be no abnormal screening studies, minimal history Presence of typical aetiology may prompt evaluation
Liver diseaseLiver failure	Multiple procoagulant and antithrombotic abnormalities Abnormal fibrinolysis Thrombocytopenia	Epistaxis Mucosal bleeding Gastrointestinal bleeding Menorrhagia Bruising Floating stools Jaundice	INR variable aPTT variable Fibrinogen variable CBC/D/Plt (thrombocytopenia) PFA-100 variable	Clotting factor activities Protein C/S activities ATIII activity vWD panel ADAMTS 13 VET	May evolve from rebalanced haemostasis into haemorrhagic state May have thrombocytopenia and dysfunction
Single ventricle right heart failure	Multiple procoagulant and antithrombotic abnormalities	Cyanosis Epistaxis Mucosal bleeding Gastrointestinal bleeding Menorrhagia Bruising Thrombosis	INR variable aPTT variable Fibrinogen variable PFA-100 normal	Clotting factor activities Protein C/S activities ATIII activity VET	May have platelet issues if hypersplenic or if asplenic PFA-100 invalid if too polycythaemic, anaemic, and/or thrombocytopenic
**Platelets**					
Renal failure	Platelet dysfunction Thrombocytopenia Anaemia	Epistaxis Mucosal bleeding Gastrointestinal bleeding Menorrhagia Bruising Dyslipidaemia Uraemia	CBC/D/Plt variable INR normal aPTT normal Fibrinogen normal PFA-100 variable	vWD panel Protein C/S activities ATIII activity Platelet aggregation VET	May be procoagulant if FVIII/vWF high, ATIII low PFA-100 invalid if too polycythaemic, anaemic, and/or thrombocytopenic
**Combined**					
Cyanosis/polycythaemia	Multiple procoagulant and antithrombotic abnormalities Thrombocytopenia	Epistaxis Mucosal bleeding Gastrointestinal bleeding Menorrhagia Bruising	CBC/D/Plt variable INR variable aPTT variable Fibrinogen variable PFA-100 variable	Clotting factor activities Protein C/S activities ATIII Platelet aggregation VET	High haematocrit may require special coagulation tubes PFA-100 invalid if too polycythaemic, anaemic, and/or thrombocytopenic

## Preoperative screening for haemostatic disorders

The potential impact on patient morbidity from previously unrecognised haemostatic disorders, whether inherited or acquired, warrants targeted preoperative screening. Concerns that arise from patient history, family history, physical examination, and laboratory haemostatic screening should prompt further investigation and considerations for preoperative haematology consultation.^17^ The International Society of Thrombosis and Haemostasis (ISTH) has developed and validated a bleeding assessment tool modified to include adults and children (ISTH-BAT) to translate the severity of bleeding into a bleeding score.^17^ The tool contains questions about severity and frequency of multiple types of bleeding events, including epistaxis, menorrhagia, cutaneous bleeding, oral and dental bleeding, gastrointestinal bleeding, postpartum bleeding, surgical bleeding, and neonatal/paediatric-specific bleeding such as cephalohaematoma, umbilical stump bleeding, and bleeding with circumcision (https://www.isth.org/page/reference_tools, accessed 28 June 2024). A score of ≥3 has been evaluated to signal a haemostatic defect in children. As vWD is the most common inherited haemostatic defect, the ISTH-BAT has been validated for this condition with a threshold to define severe bleeding ranging from ≥2 to ≥3.^18^ The largest study recruited >1200 children using a threshold of ≥3 and reported a sensitivity and specificity of 0.97, a positive predictive value of 0.48, and a negative predictive value of 0.99.^19^ Ongoing validation studies include paediatric patients in international settings (e.g. ISTH-BAT bleeding score in normal children). A tool similar to the ISTH questionnaire has been developed for neonates.^20^ However, one limitation is that the ISTH-BAT does not include elements of family history as additional clues to an inherited disorder and does not include consideration of developmental haemostasis. Limitations in clinical application are described in a recent study of a subset of 1502 consecutive adult and paediatric surgical patients with abnormal ISTH-BAT, abnormal haemostatic parameters, or both, who reported no major surgical bleeding.^21^ Another recent publication described 97 children aged <18 yr in whom the ISTH-BAT and PFA testing correlated poorly with each other and did not identify mild inherited platelet disorders.^22^

Recognising that bleeding questionnaires could be of limited benefit in a very young patient with a short personal medical history, it is unfortunate that the value of standard laboratory tests such as the activated partial thromboplastin time (aPTT) or prothrombin time (PT) to screen for inherited or acquired bleeding disorders may be limited or of no value, especially in selected patients with mild bleeding disorders (Table 1, Table 2).^21^^,^^23^^,^^24^ The aPTT is reported to be prolonged in patients with severe inherited haemophilia and vWD type 3, but one-third of the observed aPTT prolongations were as a result of FXII deficiency, which is not associated with an increased bleeding tendency.^25^ Of note, one meta-analysis also reported that the *in vitro* bleeding time measurement with the PFA-100 closure time had a high diagnostic odds ratio (110.7; 95% confidence interval 24.4–502.3, *I*^2^=0) to detect a potential risk of increased surgical blood loss or a haemostatic defect.^23^ In preterm neonates with thrombocytopenia, the PFA-100 closure time showed a better association with bleeding than the platelet count, suggesting the compensatory effect of high vWF activity in primary haemostasis of the immature neonatal coagulation system and the potential of PFA-100 closure time to guide platelet transfusions in this high-risk patient population.^26^ The PFA-100 closure time similarly provides valuable identification of the cause of bleeding disorder in heritable vWD, although it is sensitive to low haematocrit, thrombocytopenia, and other forms of platelet dysfunction.

Currently there are no universally accepted expert consensus recommendations for preoperative routine screening tests in paediatric patients who are at high risk for bleeding. The most important tool is the screening bleeding questionnaire, although response to major haemostatic challenges may be lacking in the youngest patients. Routine screening tests, such as platelet count, PT, PTT, fibrinogen, may be ordered in high-risk patients, especially those who have a positive screening questionnaire. Those who have a specific family history or condition for a bleeding diathesis require specific investigation (Table 1, Table 2) and consideration should be given to referral to a paediatric haematologist. If a platelet function abnormality is suspected, a quantitative test is indicated.

## Perioperative viscoelastic testing for haemostatic disorders

The management of haemostasis disorders in the paediatric surgery perioperative setting, as with adults, increasingly relies on the results of VET, which provides readily available data to adequately address the dynamic process of disordered coagulation often associated with trauma or major cardiac and noncardiac surgery. However, even in larger adult surgical populations, studies of patient outcomes using VET-guided transfusion practice are not entirely conclusive. Heterogeneous outcomes of VET guidance have been evaluated in meta-analyses, including a reduction in exposure to allogeneic blood products, postoperative bleeding, need for re-exploration, mortality, and others, with inconsistent conclusions.^27^^,^^28^ Although a recent meta-analysis that included only studies using the thromboelastography (TEG) device in adult cardiac surgery, trauma surgery, and liver surgery populations showed reductions in bleeding rates, length of ICU stay, and mortality, this experience may not be comparable or interchangeable with other VET devices. Unfortunately, it must be acknowledged that the multiple available VET test systems differ widely in their methods used to detect blood clotting, the assortment of activators/inhibitors used, the variety of results profiles produced, and the limited experience with sequentially emerging new devices, all of which contribute to inhomogeneity of VET data sets and challenge rigorous meta-analyses.^29^

In paediatric patients, assessing haemostatic balance is even more complex, as developmental haemostasis must be incorporated into VET interpretation and management decisions. Compared with term neonates, premature infants begin with a hypocoagulable profile, yet have clinically balanced haemostasis, and then mature to a more procoagulable state within a month of life (Fig. 1). As mentioned earlier, it appears that increased concentrations of vWF compensate for plasma coagulation factor deficiencies and platelet hyporeactivity,^30^ but current VET is insensitive to this interaction because it does not measure primary haemostasis and is relatively insensitive to von Willebrand abnormalities. Factor level maturation and platelet function effects on haemostatic balance have been reported in a systematic review of TEG and rotational thromboelastometry (ROTEM), noting accelerated initiation of coagulation, increased clot strength, and increased fibrinolysis in neonates compared with children and adults.^31^ In a recent single-centre analysis in which ROTEM reference ranges were divided into five groups ranging from 0 to <11 yr of age, the most marked changes were seen in the 0- to 12-month period, particularly for clotting time in the extrinsic pathway activation assay (ExTEM), with clear hypercoagulability, although a previous study found median shorter ExTEM clotting times at this age.^32^ Further work is needed to determine the structural/functional differences between neonatal and adult fibrinogen and the age-specific impact on VET assays.^30^^,^^32^^,^^33^ The currently most widely used systems, such as ROTEM and TEG, still lack reference values from which age-appropriate transfusion thresholds can be developed and studied. Reference values ranging from neonates to older children are available only from a small number of single- or dual-centre studies, which are not consistent with one another as mentioned above.^32^^,^^34^, ^35^, ^36^, ^37^ Nevertheless, early studies using age-adjusted internal reference ranges and transfusion algorithms with thresholds based on these results are promising, and a major published paediatric guideline (NATA) supports VET use with tailored transfusion algorithms for perioperative bleeding management in cardiac surgery.^35^^,^^38^

In a recent retrospective, small single-centre study, after establishing internal reference ranges and transfusion thresholds for ROTEM in specific age groups,^36^ the introduction of ROTEM in neonates undergoing complex cardiac surgery showed that patients in the ROTEM group received fewer platelets and cryoprecipitate than the historic pre-ROTEM cohort. One prospective cardiac surgery study compared a transfusion protocol based on routine practice *vs* an internally developed and validated ROTEM-based algorithm for which the thresholds for platelet transfusion were an ExTEM amplitude after 10 min of 30 mm and a fibrin polymerisation assay of rotational thromboelastometry (FibTEM) amplitude after 10 min of 5–10 mm.^37^ Transfusion of 10 ml kg^−1^ platelets, 15–20 ml kg^−1^ fresh frozen plasma (FFP), or both based on the VET algorithm was associated with a reduction in ICU chest tube drainage, FFP, and platelet transfusions. In a study of 139 neonates undergoing noncardiac surgery, transfusion of FFP was reduced after the introduction of age-dependent reference values for TEG into institutional transfusion algorithms.^39^

Thus, much collaborative, multidisciplinary research is required to first develop clear age-grouped reference ranges for each test system and second, based on this, establish intervention/transfusion thresholds before VET-guided haemostasis management guidelines can be developed, studied, and appropriately implemented. In addition, more research is needed to investigate the use of point-of-care VET tests to screen for or diagnose preoperative inherited coagulopathy.

## Bleeding in special paediatric conditions

Haemostatic adverse events during paediatric cardiac surgery, major noncardiac surgery, and trauma may be associated with early identifiable medical and surgical conditions.^40^ In order to study these critical preventable non-surgical bleeding events, clinicians need standardised measurement criteria. In 2019, a multidisciplinary expert consensus conference developed diagnostic criteria to define severe bleeding in critically ill children (Table 3).^41^ Thereafter, the Bleeding Assessment Scale in critically Ill Children (BASIC), a multidisciplinary physician-driven definition for bleeding severity, was validated and revealed substantial interrater reliability.^41^ Although intended for critically ill children, BASIC may also be utilised as a risk assessment tool in the dynamic setting of the operating room.

**Table 3:** Bleeding Assessment Scale for Critically Ill Children Perioperatively (BASIC Scale).^41^ PELOD-2 indicates paediatric logistic organ dysfunction score.

Any of the following criteria define severe bleeding
➢ Bleeding that leads to at least one organ dysfunction, using PELOD-2 score criteria of organ dysfunction, within 24 h of the previous assessment (if there is no previous assessment, the baseline results are presumed to be normal). The organ dysfunction should be associated with the bleeding in absence of other causes.
➢ Bleeding that leads to haemodynamic instability, defined as an increase in heart rate >20% from baseline or a decrease in blood pressure by >20% from baseline. The haemodynamic instability should be associated with the bleeding in absence of other causes.
➢ Bleeding leading to a decrease in haemoglobin >20% within 24 h. The decrease in haemoglobin should be associated with the bleeding in absence of other criteria.
➢ Quantifiable bleeding ≥5 ml kg^−1^ h^−1^ for ≥1 h (chest tube, drain)
➢ Intraspinal bleeding leading to loss of neurological function below the lesion, non-traumatic intra-articular bleeding leading to a decreased range of movement, or intraocular bleeding leading to impaired vision.

The following details considerations with respect to risk factors, prevention, and treatment specifically for cardiac, major noncardiac, and trauma surgery in the paediatric patient.

### Cardiac surgery

#### Risk factors for haemostatic bleeding

Cardiopulmonary bypass (CPB) contributions to coagulopathy have been previously described in adults,^42^ but major differences exist compared with children. Perioperative haemostasis is influenced by the degree of haemodilution that depends on patient's blood volume, baseline blood concentrations (haematocrit, coagulation factors, platelets), and also the volume and composition of CPB priming volume. Miniaturisation of the CPB circuit in neonates and children is achieved using small-volume circuit components (oxygenator, filter, tubing) and vacuum-assisted drainage. Reducing the priming volume for neonates from 325 ml to 125 ml in a single-centre retrospective study resulted in fewer transfusions in low-body-weight neonates.^43^^,^^44^ The current standard CPB priming volume for a neonatal CPB circuit in North American centres is typically 150–250 ml, and even less in selected European centres, with some centres using as little as 70 ml.^44^ Selected centres routinely achieve transfusion-free cardiac surgery even in neonates using very small priming volumes of 73–110 ml.^45^ Beyond miniaturising the circuit, the CPB priming volume composition is adapted to the patient and the type of surgery. Generally, the lower the patient's blood volume and haematocrit, the more allogeneic blood is added to the priming volume. Although fresh whole blood is used selectively, most institutions use reconstituted whole blood (red cells and FFP) for smaller patients.^46^^,^^47^ A prospective randomised trial reports that FFP priming provides higher fibrinogen concentrations and improved viscoelastic test results using TEG values before separation from CPB, but without reducing postoperative transfusions.^48^ In the randomised Albumin vs. Plasma for PaEdiAtric pRiming (APPEAR) trial, adding FFP to the CPB priming was associated with a decreased postoperative blood loss compared with a late FFP administration strategy.^49^ However, this did not translate into a difference in transfusion requirements. Because of the significant blood dilution in a large paediatric CPB circuit, activated clotting time levels may be appropriately prolonged despite decreasing heparin concentrations during CPB. It is known that protamine overdose can impair the coagulation status. A single-centre RCT demonstrated that individualised heparin/protamine management maintaining a target heparin concentration during CPB and titrating protamine dose according to the heparin concentration reduced the number of transfusions and improved clinical outcomes in infants undergoing cardiac surgery when compared with standard weight-based heparin/protamine management.^49^

**Fig 3:**
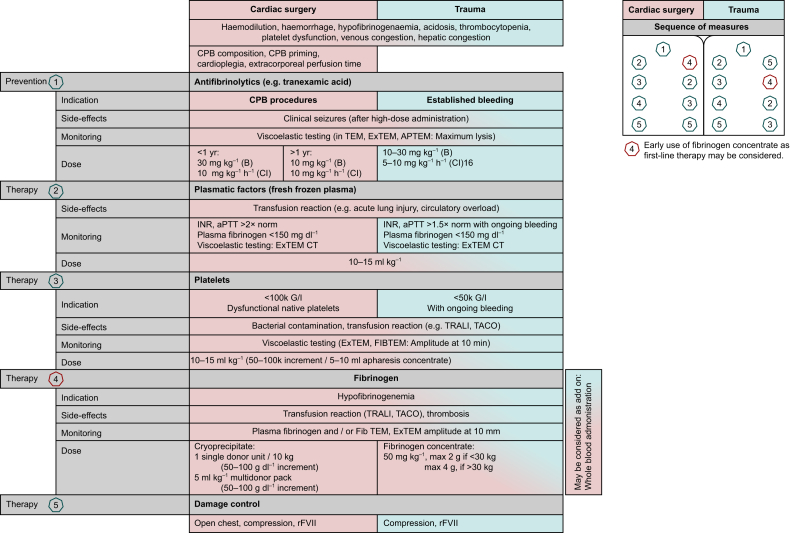
Causes, preventive measures, and treatment options of coagulopathy in cardiac surgery and trauma. aPTT, activated partial thromboplastin time; B, bolus; CI, continuous infusion; CPB, cardiopulmonary bypass; INR, international normalised ratio; TACO, transfusion-associated circulatory overload; TRALI, transfusion-associated acute lung injury.

Besides CPB volume and composition, perfusion strategy varies among institutions and impacts the coagulation system and transfusion demand. For example, a Norwood procedure by standard techniques requires periods of cardiac arrest or selective antegrade cerebral perfusion under hypothermic conditions. Besides the effects of hypothermia on the coagulation system (e.g. thrombocytopenia, platelet dysfunction, and abnormal fibrinolysis), both cooling and rewarming significantly prolong the duration of CPB, which impacts the coagulation system.^43^ Some centres use a complex perfusion technique with three arterial lines to perfuse the brain, the heart, and the distal torso.^50^^,^^51^ Using such a strategy, moderate hypothermia is suitable as the target temperature range and duration of CPB may be shortened. In a larger single-centre experience, this strategy translated into reduced transfusion requirements compared with the previously used deep hypothermic circulatory arrest approach.^50^ Given the complexity of the patient population, this practice varies from centre to centre and obtaining definitive evidence is challenging even from randomised clinical trials. Guidelines recommend the standard use of antifibrinolytic therapy in all paediatric cardiac procedures.^52^^,^^53^ Tranexamic acid (TXA) is the most commonly used agent in this context. Given the dose-dependent increased risk of perioperative seizures with its use in open heart surgery and the risk of bleeding in case of underdosing, a careful dosing scheme is important. TXA dosing suggested by recent guidelines can be seen in Fig. 3.^53^ Weight-adjusted low-dose TXA protocols have been described that target a moderate but highly effective TXA plasma concentration of 20–30 μg ml^−1^.^54^ In this protocol, a loading dose of 6.4 mg kg^−1^ was followed by an infusion of 2–3 mg kg^−1^ h^−1^. This moderate dosing should be confirmed in clinical studies with respect to safety and efficacy.

#### Treatment of haemostatic bleeding

Thrombocytopenia and platelet dysfunction are common causes of post-CPB bleeding, and transfusion requirements in paediatric cardiac surgery are influenced by CPB duration, haemodilution,^55^ and hypothermia. Few paediatric studies report the efficacy of platelet transfusion on postoperative bleeding or have compared different volumes and types of platelet products (e.g. cold-stored platelets, apheresis single donor concentrates, pooled platelet concentrates, pathogen-inactivated/-reduced platelets). Platelet transfusion recommendations are mainly guided by expert consensus, and, in part, based on adult cardiac and paediatric transfusion guidelines.^56^

Plasma coagulation factors, including fibrinogen, are usually replaced by transfusion of plasma.^57^ However, plasma is not an ideal source to provide substantial increase in low plasma fibrinogen concentrations in massive bleeding.^58^ In fact, fibrinogen is a critical coagulation factor for infants and children undergoing cardiac surgery, showing a clear relationship between intraoperative fibrinogen concentrations and postoperative blood loss.^59^ In the randomised, placebo-controlled FIBCON trial,^60^ 90 infants were randomly allocated in a 2:1 ratio to receive either fibrinogen or placebo. The trigger for administration of fibrinogen concentrate was a ROTEM FibTEM MCF value ≤6 mm. Primary endpoints were achievement of a FibTEM MCF of 8–13 mm and plasma fibrinogen concentration of 1.5–2.5 g dl^−1^ after administration of fibrinogen concentrate. Secondary endpoints were thrombosis-related complications and 24-h postoperative mediastinal bleeding. In the patients who were treated with fibrinogen concentrate, the median administered fibrinogen dose was 114 mg kg^−1^ (range 51–218 mg kg^−1^), plasma fibrinogen concentration increased from a mean of 0.91 (standard deviation [sd] 0.22) to 1.7 g L^−1^ (sd 0.41). The 24-h mediastinal drain loss after surgery was considerably lower in the fibrinogen group than in the placebo group (11.6 *vs* 17.1 ml kg^−1^) despite the fact that plasma transfusion was almost tripled in the placebo group. Five thrombotic events (8.3%) in the fibrinogen group *vs* two events (6.7%) in the placebo group were reported, all occurred at the site of femoral intravascular access which itself is a well-known risk factor for development of thrombosis, and as such, none was clearly related to fibrinogen concentrate. Of note, fibrinogen concentrate was administered during CPB, which is controversial in current practice and may explain the need for high (four-fold) doses of fibrinogen concentrate to achieve therapeutic plasma concentration. Nevertheless, the safety profile of fibrinogen concentrate in cardiac surgery, even if it is not attributable to therapy, should be the subject of further investigation in appropriate studies. Faraoni and colleagues^59^ reported that fibrinogen concentrations <150 mg dl^−1^ and FibTEM <3 mm predicted post-CPB bleeding. Cryoprecipitate is the primary source of fibrinogen replacement in the USA and UK. In other countries, fibrinogen concentrates are used. Although fibrinogen concentrate is a purified factor concentrate, despite trace amounts of FXIII, cryoprecipitate also contains large vWF multimers, FVIII, and FXIII. Considering the high incidence of acquired vWD in children with congenital heart disease, cryoprecipitate could theoretically provide more effective haemostasis compared with fibrinogen concentrate. However, several studies have compared transfusion strategies using cryoprecipitate *vs* fibrinogen concentrates in various infant, paediatric, or both CPB study designs and provided inconclusive evidence as to whether one agent is preferable to the other, so additional research is warranted.^61^, ^62^, ^63^

Four-factor prothrombin complex concentrates (4F-PCC), often combined with the fibrinogen concentrate administration, are increasingly used as a substitute for FFP in adult and paediatric patients.^64^

However, the safety profile of these potent agents has only been arbitrarily evaluated and needs further investigation in larger prospective studies. A prospective study in 15 neonates undergoing elective cardiac surgery requiring CPB has demonstrated that even a very lose dose of 4.7–14 U kg^−1^ of 4F-PCC was sufficient to enhance lag time, the peak amount, and the rate of thrombin generation.^65^ A propensity score-matched analysis in 215 children with congenital heart disease undergoing major cardiac surgery requiring CPB has demonstrated that administration of fibrinogen concentrate and 4F-PCC compared with transfusion of plasma was well tolerated and permitted haemostasis to be maintained, even in the very young.^64^ However, current guidelines recommend that 4F-PCC be used only in the context of prospective studies (Fig. 3).^38^

#### Rescue strategies in persisting severe coagulopathy

In bleeding children with massive microvascular haemorrhage and coagulopathy unresponsive to ‘standard’ treatment recombinant factor FVIIa and FVIII inhibitor bypassing agents (FEIBA) have been used as rescue therapy. However, because of the high risk of thrombotic complications, current guidelines advise against the use of these agents. Secondary chest closure with packing for local haemostatic control is a well-established strategy, especially after complex neonatal cardiac surgery when diffuse bleeding persists. Data on infection risk are inconclusive, but secondary chest closure within 3 days appears to be associated with a low risk of infection.^66^^,^^67^

### Major noncardiac surgery

#### Risk factors for haemostatic bleeding

Acquired coagulopathy associated with major paediatric surgery occurs with procedures frequently incurring high blood loss. The aetiology includes massive haemorrhage with massive transfusion and dilutional coagulopathy caused by lack of a balanced resuscitation approach. Thrombin generation, fibrinogen polymerisation, and fibrinolysis can be altered by dilutional coagulopathy, which usually occurs when lost blood is replaced with fluids that do not contain adequate coagulation factors.^68^ Contributing risk factors for exacerbating coagulopathy in the paediatric patient undergoing major surgery with clinically significant blood loss include hypotension, acidosis, hypothermia, and fibrinolysis.

Hypofibrinogenaemia and fibrinogen polymerisation disorder is commonly one of the first derangements with a subsequent imbalance in coagulation factors II, VII, IX, and X.^68^, ^69^, ^70^, ^71^, ^72^ With blood loss less than 50% of the total blood volume, platelet function is generally preserved. The mechanism of coagulopathy in major surgery is usually directly related to a deficit in haemostatic coagulation factors and dilutional coagulopathy.

The multifactorial causes of acquired coagulopathy necessitate the use of a fast and reliable test to detect it as early possible and to guide the individual bleeding management.^73^ The most recently published guideline recommends performance of standard plasma coagulation testing, if no viscoelastic tests are available.^35^

#### Prevention and treatment of haemostatic bleeding

Antifibrinolytic agents are considered the most important therapeutic modality in the prevention/treatment of haemostatic bleeding in paediatric major noncardiac surgery, including craniofacial, maxillofacial, major orthopaedic, and neurosurgery (Fig. 3).^74^^,^^75^ Published RCTs in paediatric major surgery consistently report that children allocated to receive an antifibrinolytic agent compared with placebo have a reduction in blood loss and transfusion with a favourable safety profile.^76^, ^77^, ^78^, ^79^, ^80^ Expert consensus concludes that use of antifibrinolytics is efficient and safe in children undergoing major surgery.^75^^,^^81^

Although plasma transfusions are widely used to prevent or treat acquired coagulopathy in children, the beneficial effect was never proved during major surgery nor in critically ill children.^82^^,^^83^ In addition, there is strong evidence that transfusion-related side-effects are associated with increased morbidity and mortality in children, and as such, a more tailored approach using targeted bleeding management may be beneficial.^84^^,^^85^

### Trauma

#### Risk factors for haemostatic bleeding

The underlying mechanisms for trauma-induced bleeding in children compared with adults are characterised by a higher rate of blunt and non-accidental trauma, including brain injuries.^86^ Although the aetiology of adult trauma coagulopathy has been hypothesised to be attributed to tissue factor-related increase in thrombin generation, protein C activation, hypoperfusion, and/or hyperfibrinolysis, the mechanisms in paediatric trauma patients remain not widely studied. However, severe hypofibrinogenaemia (<100 mg dl^−1^) is a frequent finding in paediatric trauma patients (>20%).^87^ Life-threatening coagulopathy is present in almost all patients with an injury severity score >25, hypotension, hypothermia, and acidosis. Severe traumatic brain injury (TBI) is a major contributor to the development of trauma-induced coagulopathy (TIC), occurring in 42–44% of severe TBI cases,^88^^,^^89^ and an important contributor to mortality.^90^ The incidence of TIC in paediatric trauma patients ranges widely from 10% to 77%,^91^ which is likely to be because of the lack of standardised TIC definitions, variable coagulation testing, and the need for validation of specific thresholds. Notably, hypercoagulable and hypocoagulable states occur in paediatric TBI.^92^ Hypercoagulability promotes ischaemic lesions, which may worsen outcomes.^91^ Severe hypofibrinogenaemia and thrombocytopenia are not uncommon,^86^ although the degree of thrombocytopenia does not always correlate with bleeding severity.^93^

#### Prevention and treatment of haemostatic bleeding

Evidence supporting routine antifibrinolytic administration in paediatric trauma is sparse and roughly extrapolated from the adult trauma population together with use in paediatric cardiac and noncardiac surgery (Fig. 3).^74^^,^^75^ In a recent secondary analysis of the MAssive Transfusion epidemiology and outcomes In Children (MATIC) study dataset, a prospective international observational study of children with life-threatening bleeding events, the use of antifibrinolytics in children with life-threatening haemorrhage was independently associated with improved 6-h and 24-h survival in bleeding children supporting the early administration of the use of antifibrinolytics in paediatric patients with life-threatening haemorrhage.^94^ A small pilot prospective randomised trial evaluated TXA in children with severe traumatic haemorrhage (TIC-TOC)^113^ and compared TXA 15 mg kg^−1^ bolus dose 2 mg kg^−1^ h^−1^ *vs* TXA 30 mg kg^−1^ bolus dose, followed by 4 mg kg^−1^ h^−1^ infusion *vs* placebo. No difference was found in Paediatric Quality of Life score, transfusion requirements, or adverse effects, although the study was not powered for clinical outcomes.

Although the risk of thrombotic complications with higher TXA dosing regimens and the risk of trauma-associated fibrinolytic shutdown may be less in the paediatric patients compared with adults, further studies are needed in paediatric patients to better test the benefit–risk profile of dosage regimen based on the pharmacokinetic and pharmacodynamic profiles of TXA. Future focus in this area should incorporate an individualised approach based on the point-of-care assessment of the patient's unique dynamic fibrinolytic profile.^95^ In conclusion, the administration and dose of TXA in paediatric trauma-associated haemorrhage is recommended based on extrapolation of a favourable risk–benefit ratio in a dose of 10–30 mg kg^−1^ loading dose, best given within 3 h of the onset of haemorrhage, followed by a continuous infusion of 5–10 mg kg^−1^ h^−1^.^53^^,^^74^

## Goal-directed treatment of severe haemostatic bleeding associated with major surgery, trauma, or both

Management of acquired coagulopathy in paediatric trauma depends on the aetiology. Coagulation optimisation using multimodal strategies includes treatment with goal-directed massive haemorrhage/transfusion protocols (Table 4), haemostatic agents, and point-of-care testing to guide diagnosis and management to treat or prevent the haemostatic coagulopathy in the bleeding child and not to correct the specific laboratory derangement.^96^ Damage control resuscitation and activation of critical bleeding/massive haemorrhage protocols are the first strategies used.^97^ The aims of damage control resuscitation are detailed in Table 5. Damage control resuscitation aims to limit blood loss and prevent coagulopathy by combining resuscitation to maintain blood pressure and perfusion, early airway control to maintain oxygenation, and early and balanced use of blood products and other haemostatic agents to achieve haemostasis and prevent unnecessary transfusion, hyperkalaemia, hypothermia, and acidosis. Furthermore, emergency allogenic blood transfusion management should include evidence-based and goal-directed strategies to transfuse indicated haemostatic products and avoid unnecessary allogeneic exposures. Special consideration must be made for severely injured paediatric patient with haemorrhagic shock, as TIC can quickly develop. Rapidly initiated resuscitation should be similarly managed with age-appropriate goal-directed interventions, including transfusion. The benefit of pre-hospital transfusion with red cells was shown in a recent retrospective study from a nationwide trauma database that suggested improved outcomes in transfused severely injured children.^98^

**Table 4:** Goal-directed management strategies for paediatric coagulopathy in major trauma/cardiac and noncardiac surgery.

1. To prevent/stop/control the bleed and correct aetiology for haemostatic derangement.
2. To maintain haemodynamic stability, end-organ perfusion, and oxygen delivery.
3. To optimally transfuse specific allogeneic haemostatic blood products (such as fresh frozen plasma, platelets, cryoprecipitate) using balanced goal-directed therapy and to reduce the side-effects associated with blood component transfusion.
4. To minimise exposure and avoid over-transfusion (causing haemodilution and dilutional coagulopathy) with appropriate utilisation blood conservation strategies, such as restrictive transfusion, antifibrinolytics, and/or recombinant components (such as fibrinogen concentrate and prothrombin complex concentrates).
5. To optimise individualised coagulopathy management guided by point-of-care tests such as laboratory testing and viscoelastic testing to maintain the balance between bleeding and thrombosis.

**Table 5:** Ten basic universal components of a paediatric massive haemorrhage protocol.

1. Activate rapid damage control resuscitation. Time matters.
2. Trigger massive haemorrhage protocol activation with goal-directed management.
3. Massive transfusion protocol implementation with early balanced resuscitation can improve survival.
4. Administer blood products and crystalloids on a per kilogram basis and avoid fluid overload and haemodilution.
5. Goal-directed coagulation management is preferred using point-of-care testing to guide management.
6. Tranexamic acid should be administered early in the bleeding paediatric surgical or trauma patient.
7. To prevent the lethal triad of coagulopathy, acidosis, and hypothermia.
8. Avoid premature administration of frozen plasma and platelets.
9. Cryoprecipitate or fibrinogen concentrate is first line for fibrinogen replacement.
10. Consider using recombinant factor concentrates in settings where frozen plasma is unavailable.

Expert consensus recommends that paediatric massive haemorrhage protocols incorporating a massive transfusion protocol (MTP) based on age or weight should be readily accessible and available. Massive haemorrhage protocol activation typically use the following criteria: (1) haemodynamic instability with blood loss >40 ml kg^−1^ within 4 h or blood loss >20% total blood volume and anticipated ongoing bleeding; (2) severe thoracic, abdominal, pelvic, or multiple long bone trauma, or major head trauma; and (3) major gastrointestinal or surgical bleeding. Multiple goal-directed paediatric MTPs are available, such as Australian Patient Blood Management Guidelines: Module 6 | Neonatal and Paediatrics, the Ontario Transfusion Coordinator program (ONTRAC), and Boston Children's Hospital published by Society for the Advancement of Blood Management (SABM). Several guidelines incorporate VET into the goal-directed decision tree for coagulopathy management.^99^ Key recommendations for paediatric massive haemorrhage protocols are outlined in Table 5.

Although expert recommendations are published,^100^ there is insufficient high-quality evidence to recommend specific transfusion strategies to direct plasma or platelet transfusion. MTPs are generally designed to avoid coagulopathy that historically arose from transfusion of predominantly red cells by supplying fixed ratio, rapidly prepared and delivered red cells, plasma, and platelets in an institution-specific ratio; cryoprecipitate is variably also included. Although evidence regarding the utility, feasibility, and improvement in paediatric patient outcomes is weak and largely extrapolated from adults, high-quality evidence exists that using fixed ratios of blood products may improve outcome in coagulopathic military and civilian adult trauma patients; however, it is unknown how this translates to paediatric trauma whose mechanism of injury is different from adults.^101^^,^^102^ Two recent expert consensus conferences, the Pediatric Critical Care Transfusion and Anemia Expertise Initiative (TAXI) and Transfusion-Anemia eXpertise Initiative-Control/Plasma and Platelets in Critically Ill Children Avoidance of Bleeding (TAXI-CAB), developed recommendations for massive haemorrhage in children. Both advocate a resuscitation strategy of red blood cells–plasma–platelets in ratios of 1:1:1 or 2:1:1 in critically ill children with haemorrhagic shock. It is cautioned that a fixed ratio-driven approach may lead to inappropriate or unnecessary transfusions.^103^ A fibrinogen replacement source is not included in many MPTs. The MATIC investigators recently reported a retrospective cohort study showing that even if MTPs are feasible in paediatric patients, they are associated with increased blood product transfusion with no increased survival in children.^104^ Timing of initiation in this study was variable and often delayed, potentially contributing to these findings.

Whole blood haemostatic resuscitation in paediatric trauma has been suggested as a feasible and safe alternative to component therapy, given its role in the adult trauma setting. Only small single-centre studies suggest that efficacy is associated with decreased transfusion requirements and ventilation days in paediatric trauma patients compared with component therapy.^105^^,^^106^ However, a recent larger analysis of the Trauma Quality Improvement Program database compared 109 paediatric patients who received whole blood transfusions with 281 patients who received conventional therapy.^107^ Patients with whole blood transfusions received lower total volumes and transfusion burden, but clinical outcomes were similar.

Expert consensus recommends that a minimum platelet count of 50 000 cells μl^−1^ should be maintained for trauma patients with ongoing bleeding. The transfusion of platelet concentrates carries the highest risk of side-effects of all allogeneic blood products (bacterial contamination of platelet components has been one of the most common cause of transfusion-related deaths in the USA and Europe (please see also: Summary of the 2019 annual reporting of serious adverse reactions and events for blood and blood components - European Commission; europa.eu)^108^ and should be performed cautiously. Strategies that consider underlying coagulopathy, using standard laboratory coagulation tests or viscoelastic assays, may allow individualised therapies and are likely to improve outcomes.^103^

Clinical guidelines have historically suggested replacing coagulation factor deficiency with FFP when ongoing clinical bleeding and the international normalised ratio and aPTT are 1.5 times normal. The decision to transfuse FFP would ideally be based on VET testing, but such systems are currently insufficiently evaluated in this population. FFP transfusion volume should be calculated based on weight and desired improvement in coagulation indices (Fig. 3).^99^

Acquired fibrinogen deficiency is the leading determinant in developing coagulopathy during critical bleeding resuscitation after trauma or major surgery.^108^^,^^109^ Fibrinogen is the first coagulation factor to fall to critically low concentrations during major haemorrhage as a result of dilution and hyperfibrinolysis.^68^ Although acquired hypofibrinogenaemia treatment traditionally was cryoprecipitate transfusion, currently purified fibrinogen concentrate is increasingly used based on Clauss fibrinogen and VET results as cryoprecipitate has been withdrawn from most European countries owing to potential immunological reactions and infectious agent transmission risks as a multidonor product. A recent propensity-weighted retrospective study of 1948 patients from the Paediatric Trauma Quality Improvement Program found that patients who received cryoprecipitate during massive transfusion had significantly lower 24-h mortality than patients who did not.^110^ A retrospective double-centre study at American College of Trauma-verified Level I and Level II paediatric trauma centres included 64 patients with blunt solid organ injuries.^111^ At presentation to the emergency department, VET-based haemostatic resuscitation with platelets, cryoprecipitate, or both was initiated. Tetrachoric correlations and regression modelling were used to correlate TEG-guided resuscitation with clinical outcomes. Patients showed better outcomes in terms of length of hospital stay and mortality. The data may demonstrate the importance of obtaining rapid results using point-of-care diagnostic tools that provide the basis for early individualised and targeted therapy. Alternatively, intraoperative substitution with fibrinogen concentrate can effectively treat fibrinogen deficiency.^112^ Clinical studies with sufficient efficacy to perform a safety analysis of this agent in adults or children are lacking. There is no evidence yet supporting the efficacy, safety, or dosing of PCCs in paediatric patients.^96^ Acquired FXIII deficiency appears prevalent even in major paediatric surgery, but no data exist on paediatric FXIII supplementation. In paediatric noncardiac perioperative management, there are insufficient data to make any evidence-based recommendations concerning the efficacy of rFVIIa as prophylactic, routine, or rescue therapy in paediatric perioperative bleeding management.

## Conclusions

The diagnosis and management of severe perioperative coagulopathy in paediatric patients is challenging and complex. Prospective randomised trials in paediatric patients are difficult to conduct, further compromising the evidence. The recent development of BASIC can be considered a step towards improving the evidence. Standardising the definition of major bleeding and consolidating data into large databases may help identify areas where further research is needed and probably also help in planning more extensive prospective multicentre studies. Regarding management, the TAXI-CAB expert consensus recommendations can be useful to guide individualised goal-directed patient care.

In cardiac surgery, the patients have a high inherent risk of diseases possibly underlying a coagulation disorder in addition to post-CPB haemostatic issues. Whether undergoing cardiac or noncardiac surgery, acquired hypofibrinogenaemia and vWD have a high prevalence and impact bleeding, which must be proactively considered and appropriately screened to identify. In this context, screening with platelet function analysers (e.g. the PFA-100 closure time) may be considered in specific high-risk patients. High-quality evidence is lacking for haemostatic decision management in children with acquired coagulopathy during and after trauma or major surgery. Future research must include high-quality studies evaluating the risk–benefit ratio in managing coagulation abnormality *vs* the risk–benefit ratio of haemostatic blood product transfusion, comparative trials evaluating management strategies to determine the optimal dose, timing, and order for haemostatic blood component transfusion administration, and other interventions to treat the coagulopathic paediatric patient*.* In the high-risk settings (e.g. trauma, cardiac surgery, or major noncardiac surgery), novel goal-directed therapeutic approaches currently resemble those in adult patients focused on timely diagnosis of coagulopathy using VET and timely and targeted replacement of coagulation factors by administration of factor concentrates. However, differences in regulatory therapeutic approval between adults and children (including international differences for pharmacologic adjuncts and laboratory testing devices) have contributed to differences in therapeutic strategies. Publication of inhomogeneous experience further limits the establishment of common data elements and hampers the extrapolation of valid evidence from prospective studies. However, particularly in neonates and small infants, validation of normal reference values in VET and defining age-grouped VET-based transfusion thresholds is a prerequisite for further therapy studies.

## Authors contributions

Conceptualisation: GE, AK, MS, JHL, SMG, TH.

Methodology: GE, AK, MS, JHL, SMG, TH.

Formal analysis: GE, AK, MS, JHL, SMG, TH.

Writing - original draft preparation: GE, AK, MS, JHL, SMG, TH.

Writing - review and editing: GE, AK, MS, JHL, SMG, TH.

Have read and agreed to the published version of the manuscript: all authors.

## Funding

There was no funding for this manuscript.
